# Integrating machine learning and neural networks for new diagnostic approaches to idiopathic pulmonary fibrosis and immune infiltration research

**DOI:** 10.1371/journal.pone.0320242

**Published:** 2025-04-24

**Authors:** Yali Guo, Qian Jin, Yi Kang, Wenwen Jin, Ying Liu, Qian Chen, Jian Liu, Yu guang Wang

**Affiliations:** 1 Department of Respiratory Medicine, Beijing Hospital of Traditional Chinese Medicine, Affiliated to Capital Medical University, Beijing, China; 2 Beijing University of Chinese Medicine, Beijing, China.; Showa University Fujigaoka Hospital, JAPAN

## Abstract

**Background:**

Idiopathic pulmonary fibrosis (IPF) is an interstitial lung disease with a fatal outcome, known for its rapid progression and unpredictable clinical course. However, the tools available for diagnosing and treating IPF are quite limited. This study aims to identify and screen potential biomarkers for IPF diagnosis, thereby providing new diagnostic approaches.

**Methods:**

We choosed datasets from the Gene Expression Omnibus (GEO) database, including samples from both IPF patients and healthy controls. For the training set, we combined two gene array datasets (GSE24206 and GSE10667) and utilized GSE32537 as the test set. We identified differentially expressed genes (DEGs) between IPF and normal tissues and determined IPF-related modules using Weighted Gene Co-expression Network Analysis (WGCNA). Subsequently, we employed two machine learning strategies to screen potential diagnostic biomarkers. Candidate biomarkers were quantitatively evaluated using Receiver Operating Characteristic (ROC) curves to identify key diagnostic genes, followed by the construction of a nomogram. Further validation of the expression of these genes through transcriptomic sequencing data from IPF and normal group animal models. Next, we conducted immune infiltration analysis, single-gene Gene Set Enrichment Analysis (GSEA), and targeted drug prediction. Finally, we created an artificial neural network model specifically for IPF.

**Results:**

We identified ASPN, COMP, and GPX8 as candidate biomarker genes for IPF, all of which exhibited Area Under the Curve (AUC) above 0.90. These genes were validated by RT-qPCR. Immune infiltration analysis revealed that specific immune cell types are closely related to IPF, suggesting that these immune cells may play a significant role in the pathogenesis of IPF.

**Conclusion:**

ASPN, COMP, and GPX8 have been identified as potential diagnostic genes for IPF, and the most relevant immune cell types have been determined. Our research results propose potential biomarkers for diagnosing IPF and present new pathways for investigating its pathogenesis and devising novel therapeutic approaches.

## Introduction

Idiopathic pulmonary fibrosis (IPF) represents a chronic, relentless, and ultimately fatal pulmonary affliction, distinguished by the presence of irreversible fibrosis within the lung parenchyma. Despite advancements in medical research and therapeutic strategies, the prognostic outlook for individuals afflicted with IPF continues to be unfavorable, with the median life expectancy estimated at a span of approximately three to five years post-diagnostic confirmation [1]. The underlying causes of IPF remain elusive, and despite advances in understanding its pathogenesis, the disease continues to present significant diagnostic and therapeutic challenges [2,3]. Accurate and early diagnosis is crucial for managing IPF and improving patient outcomes, but conventional diagnostic methods, such as high-resolution computed tomography (HRCT) and histopathological examination, have limitations in sensitivity and specificity [4].

In recent years, advancements in the realms of machine learning and artificial intelligence (AI) have paved novel pathways within the sphere of medical diagnostics. Neural networks, a subset of AI, have shown great promise in analyzing complex medical data and identifying patterns that may not be apparent to human observers [5].

These models have the potential to enhance diagnostic accuracy and provide insights into disease mechanisms.

The immune system exerts a pivotal influence on the pathogenic mechanisms underlying IPF [6]. Various studies have demonstrated altered immune cell infiltration and activity in the lungs of IPF patients [7]. Nevertheless, the precise nature and implications of these immune alterations are not fully understood. Elucidating the nuances of immune infiltration in IPF may offer significant insights, thereby potentially informing the development of targeted therapeutic strategies and enhancing the clinical management of patients [8].

This study aims to construct a neural network diagnostic model for IPF and investigate the characteristics of immune infiltration associated with the disease. Additionally, we aim to analyze the immune cell infiltration characteristics in IPF tissues, with the goal of identifying specific immune cell types and elucidating their roles in the pathogenesis of IPF. This investigation yields valuable insights into the identification of potential therapeutic targets and furthers the comprehension of the intricate pathophysiological mechanisms driving the disease process.

## Methods

### Data acquisition and preprocessing

Gene expression information was retrieved from the gene expression omnibus (GEO) repository. We utilized datasets GSE24206 and GSE10667 as train set, which were combined using the R package “ComBat” to eliminate batch effects [9]. These datasets comprise 21 control samples and 48 IPF samples. All samples underwent standardization to ensure data consistency and comparability for subsequent analysis [10]. In order to evaluate the dependability of the neural network model’s predictive accuracy and consistency, dataset GSE32537 was employed as an independent test set, containing 50 control samples and 167 IPF samples. The selection and preprocessing of these datasets ensure the accuracy and reliability of model training and test.

### Differential gene, WGCNA and enrichment analysis

Differential expression analysis between the normal group and the IPF group was performed using the limma package in R Studio. Then, genes with a |log-fold change (FC)| > 1 and a Benjamini and Hochberg adjusted with p-value < 0.05 were selected as differentially expressed genes (DEGs). Volcano plots and heatmap were generated using the ggplot2 and heatmap packages, respectively, to display the expression of DEGs [11]. Subsequently, we utilized weighted gene co-expression network analysis (WGCNA), a robust systems biology method, to identify modules most strongly associated with IPF [12]. By integrating WGCNA results with DEG analysis, we identified a robust collection of IPF-associated genes. To elucidate the biological processes and pathways implicated in IPF pathology, we conducted comprehensive gene ontology (GO) and kyoto encyclopedia of genes and genomes (KEGG) enrichment analyses. Leveraging the R packages “clusterProfiler”, “org.Hs.e.g.,db”, and “DOSE” facilitated thorough exploration of functional annotations and pathway mappings [13,14].

### Identification and test of IPF predictive markers

Least absolute shrinkage and selection operator (LASSO) and random forest (RF) were used to identify potential diagnostic biomarkers. LASSO is a powerful feature selection method that simultaneously performs variable selection and regularization. The regularization parameter λ was selected through 10-fold cross-validation to optimize model performance and reduce overfitting. Random Forest is an ensemble learning method that constructs multiple decision trees and combines their predictions to improve accuracy and robustness. The “glmnet” package was used to perform LASSO analysis. The “randomForest” package was used to perform RF analysis [15,16]. These algorithms were employed to screen candidate diagnostic biomarkers.

### Experimental verification

#### Animal experiment.

A cohort of twenty-four specific pathogen-free (SPF) grade male C57BL/6J mice, characterized by a weight range of 20±2 grams and an age bracket of 6–8 weeks, was procured from Sibef Biotechnology Co., LTD. (Beijing). These animals were housed in the SPF animal laboratory at Beijing University of Chinese Medicine, subjected to a standardized light/dark cycle of 12 hours each. The environmental conditions were meticulously regulated, with the ambient temperature maintained at (24±2) °C and the relative humidity controlled within the range of 40% to 70%. The experimental procedures were sanctioned by the Animal Research Ethics Committee of Beijing University of Chinese Medicine, with the ethical approval reference number: BUCM-2023092204–3191. During the experiment, all mice underwent procedures while under anesthesia. Anesthesia was induced using a 1% pentobarbital sodium solution, administered intraperitoneally at a dosage of 50 mg/kg. The model group received a single intratracheal instillation of bleomycin(BLM) (5 mg/kg) to induce lung fibrosis, while the control group received an equivalent volume of saline. After 28 days, the mice died from cervical dislocation following pentobarbital sodium anesthesia. Pentobarbital sodium was selected due to its rapid onset and minimal distress to the animals. To minimize potential pain and suffering, after the intratracheal administration of bleomycin, the mice were placed in a warm, quiet environment for recovery, with sufficient food and water provided. During and after the modeling procedure, the mice were closely monitored for changes in their mental state, body weight, respiratory status, and coat color to detect any abnormalities promptly. The entire experimental procedure was reviewed by the Animal Ethics Committee to ensure that the mice were treated in accordance with applicable welfare policies. Transcriptomic analysis was performed to assess mRNA expression levels in lung tissues from both groups using microarray technology.

#### Cell culture.

The human embryonic lung fibroblast cell line MRC-5 was procured from Wuhan Pusainuo Life Technology Co., Ltd. The cells were cultured in Minimum Essential Medium (MEM) supplemented with 10% fetal bovine serum at a temperature of 37°C in an atmosphere containing 5% carbon dioxide. Upon reaching confluence, the cells were allowed to adhere for a period of 24 hours. Subsequently, the experimental groups were established, including a control group and a transforming growth factor-beta (TGF-β) treated group at a concentration of 10 ng/mL. Each group consisted of triplicate wells to ensure experimental reliability, and the incubation was continued for an additional 48 hours.

#### RNA extraction and real-time quantitative PCR (RT-qPCR).

Total RNA extraction was performed utilizing a commercial total RNA extraction kit, followed by reverse transcription into complementary DNA (cDNA) under stringent conditions: an initial incubation at 50°C for 15 minutes, succeeded by a denaturation step at 85°C for 5 minutes. The PCR reaction mixture was assembled devoid of polymerase, comprising 0.2 μL of each forward and reverse primer, 1 μL of cDNA sample, 3.6 μL of enzyme-free sterile water, and 5 μL of fluorescent detection reagent. The amplification protocol entailed an initial pre-denaturation phase at 95°C for 30 seconds, subsequent cycling with denaturation at 95°C for 10 seconds, annealing at 60°C for 30 seconds, for a total of 40 cycles, and a melting curve analysis consisting of phases at 95°C for 15 seconds, 60°C for 60 seconds, and a final phase at 95°C for 15 seconds. Glyceraldehyde-3-phosphate dehydrogenase (GAPDH) served as an endogenous control to normalize the expression data, and the relative quantification of gene expression across various treatment groups was ascertained employing the 2-ΔΔCt comparative method. The selection of GAPDH as the reference gene was based on its stability and reliability. The sequences of the primers used are as follows: GAPDH (forward, 5’-GTCAAGGCTGAGAACGGGAA-3’ and reverse, 5’-AAATGAGCCCCAGCCTTCTC-3’), Fn (forward, 5’-CCGGGACTCAATCCAAATGC-3’ and reverse, 5’-TCCGTAGGTTGGTTCAAGCC-3’), COL-1(forward, 5’-CAATGTGGTTCGTGACCGTG-3’ and reverse, 5’-TGTTCTCGATCTGCTGGCTC-3’), COMP (forward, 5’-CCCAGAAGAACGACGACCAA-3’ and reverse, 5’- CCATCGCCATCACTGTCCTT-3’), ASPN (forward, 5’- AACAAGAGAGCCAAGAAGCCA-3’ and reverse, 5’-ATGTTGGTTGGGACTGAGGTC-3’), GPX8 (forward, 5’-ACATTCAGGTGTCCCTTCGG-3’ and reverse, 5’-AGGTAGCAGCACATCAGCAA-3’).

#### Immune infiltration analysis.

Immune infiltration analysis was conducted using the SangerBox platform (http://www.sangerbox.com/) [17]. The CIBERSORT algorithm was used to evaluate the proportions of various immune cells in the IPF and control samples. A heatmap was constructed using the “corrplot” R package to illustrate the quantitative relationships between different immune cells. A *p*-value < 0.05 was considered statistically significant between the two groups. Furthermore, the “ggplot2” package was used to analyze the correlation between the expression of key genes and the proportions of immune cells.

#### Gene set enrichment analysis and drug sensitivity analysis.

Single-gene GSEA analysis was conducted using the SangerBox platform [17]. Samples were stratified into high-expression (≥50%) and low-expression (<50%) groups based on gene expression levels. A selection of terms from the ‘c2.cp.kegg.v7.4.symbols.gmt’ file was extracted from the Molecular Signatures Database, reference number 18, facilitating the evaluation of pathways and molecular mechanisms [18]. To identify additional drug candidates targeting biomarkers for treating IPF, drug sensitivity analysis was performed. Gene expression data and drug sensitivity data were obtained from the CellMiner database [19]. R packages “impute”, “limma”, “ggplot2”, and “ggpubr” were employed for drug sensitivity analysis.

#### Single-cell RNA Sequencing analysis.

The dataset for human lung fibrosis was sourced from the Single Cell Portal (https://singlecell.broadinstitute.org/single_cell), focusing on scRNA-seq data relevant to lung fibrosis. To enhance the robustness of the results, we also performed an analysis based on the IPF Cell Atlas (http://ipfcellatlas.com/). The IPF Cell Atlas is a single-cell data resource focused on IPF. It integrates single-cell datasets from multiple studies and provides standardized visualization tools.

#### Artificial neural network-based IPF classification model.

We initially converted the expression data of DEGs into a gene scoring table based on their expression levels. Specifically, for each gene within the given sample, we compared its expression value to the median expression across all samples. For upregulated genes, if the expression value was greater than 0, we assigned a value of 1; otherwise, it was assigned 0. For downregulated genes, if the expression value was lower, we assigned 0; otherwise, 1. IPF was designated as the outcome variable, where IPF was coded as 1 and Normal as 0. Subsequently, we employed the neural network package in R to visualize the constructed gene scoring table through an ANN model [20]. In terms of model parameters, we configured five hidden layers. To mitigate overfitting and optimize model performance, we utilized the Caret package in R for 5-fold cross-test.

### Statistical Analysis

All data calculations and statistical analyses were performed using R (version 4.3.0) and GraphPad Prism (version 9.5.1) software. The predictive performance of the diagnostic model was evaluated using receiver operating characteristic(ROC) curves and area under the curve(AUC). Pearson correlation analysis was used to assess the correlation between variables. In addition, unpaired t-tests were used to assess the differential expression of genes. All *p*-values were bilateral, and *p*-value < 0.05 was deemed statistically significant.

## Results

### DEGs screening

**Fig 1:**
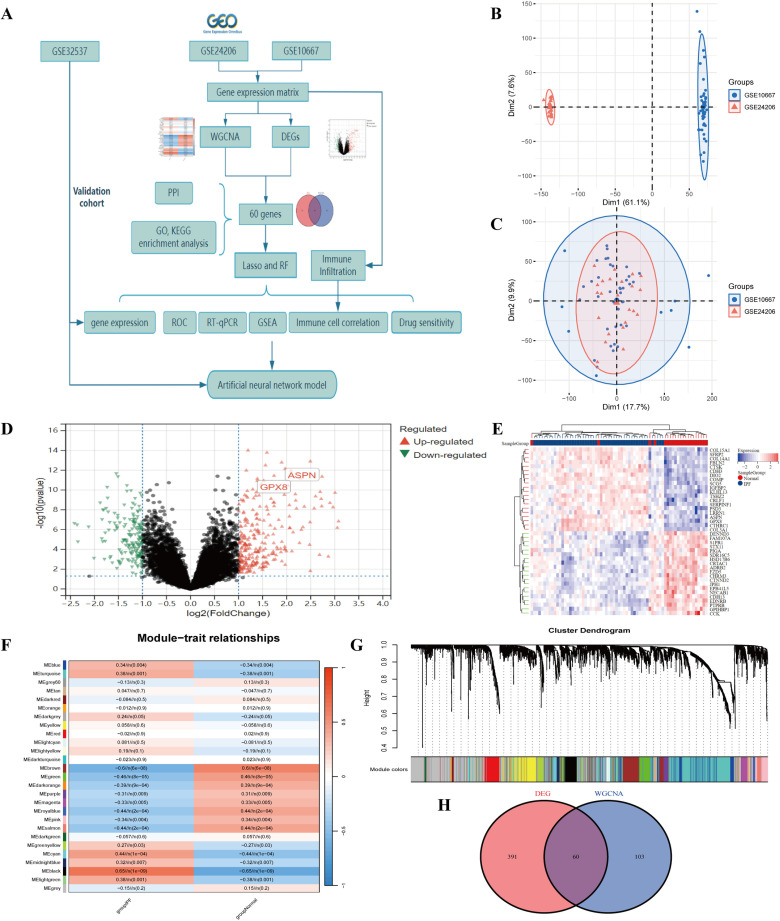
Genes differentially expressed between the IPF and normal groups. (A) Flowchart of the research. (B) PCA plot before batch effect correction; (C) PCA plot after batch effect correction. (D)Volcanic map for differential expression analysis of train set. (E) Heatmap for differential expression analysis of train set. (F) Weighted correlation network analysis of train set. (G)A gene clustering tree with multiple divided modules. Different clusters were attached with different colours. (H)Venn of DEGs and WGCNA.

Fig 1A illustrates the schematic flowchart of the study’s methodology, providing a visual overview of the research process. We first obtained the IPF dataset (GSE24206 and GSE10667) from GEO database as train set. After merging and correcting for batch effects, the data showed improved consistency and comparability across different samples. Principal component analysis (PCA) diagrams, both pre- and post-correction for batch effects, exhibited a marked decrease in variability attributable to batch differences(Fig 1B and 1C). We identified 451 DEGs in the IPF group compared to the normal group, with 277 upregulated and 174 downregulated(Fig 1D, 1E and S1 Table).

### Construction of WGCNA network and identification of IPF-related module

To determine the association of potential gene modules with IPF, we conducted WGCNA on all candidate genes from the training set. This analysis identified ten distinct modules (Fig 1F and 1G). After the analysis of the positive correlation coefficients, module black was screened out in the train set (S2 Table ).To identify co-expressed genes between WGCNA-derived hub genes and DEGs, we screened 60 overlapping genes as candidate hub genes (Fig 1H).

### PPI network construction and GO and KEGG enrichment analysis

**Fig 2:**
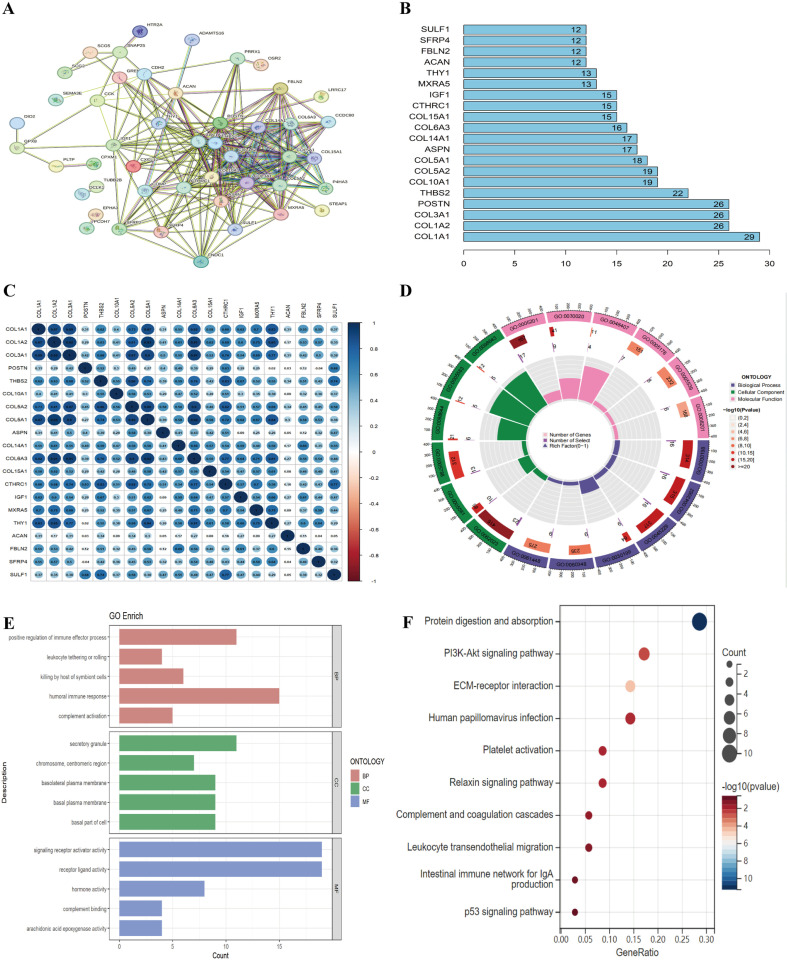
Functional enrichment analysis of DEGs. (A) PPI network. (B)Top 20 hub genes. (C) Correlations between top 20 hub genes. (D-E) GO terms of DEGs. (F) KEGG pathway analysis of DEGs.

Employing the online resource STRING, we successfully established a network depicting interactions among key genes that overlap, focusing on their protein-protein interconnections(Fig 2A). Subsequently, a visualization was carried out for the top 20 genes that were ranked highest, followed by a correlation analysis focusing on this select group of central genes (Fig 2B and 2C). Further exploration into the intrinsic functions and pathways of the 60 intersecting genes was undertaken through the execution of GO and KEGG analyses, aiming to elucidate their fundamental roles within the biological context (Fig 2D and 2F). GO enrichment analysis delineated that the intersecting genes predominantly influence the biological processes, encompassing the humoral immune response, the activation of the complement system, and the positive modulation of immune effector processes. KEGG enrichment analysis showed that the overlapping genes mainly affect PI3K AKT pathway, leukocyte transendothelial migration, and complement and coagulation cascades. The findings imply a potential role of the immune response in the etiology and progression of IPF.

### Identification of the diagnostic markers for IPF

**Fig 3:**
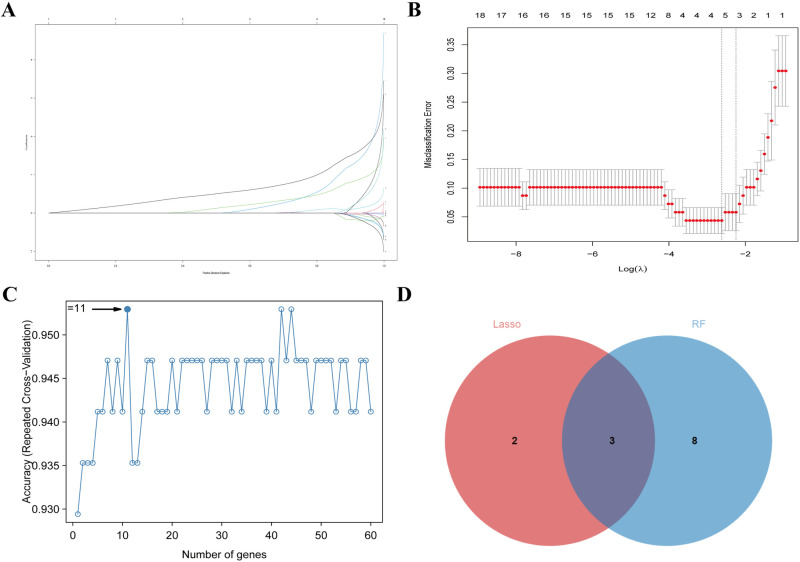
Identification of diagnostic biomarkers for IPF. (A-B) Lasso regression analysis identified five potential biomarkers for IPF. (C) RF algorithm identified eleven genes with diagnostic relevance. (D) Venn graph displaying 3 diagnosis biomarkers shared by LASSO and RF.

Utilizing two sophisticated machine learning algorithms, namely Lasso regression and the RF classifier, we endeavored to identify a panel of diagnostic biomarkers for IPF. The Lasso regression algorithm identified 5 potential biomarkers (Fig 3A and 3B), while the RF algorithm identified 11 genes with diagnostic potential (Fig 3C). The intersection of two methods revealed 3 diagnostic biomarkers: ASPN, COMP, and GPX8 (Fig 3D).

### Diagnostic power and expression of three candidate biomarkers

**Fig 4:**
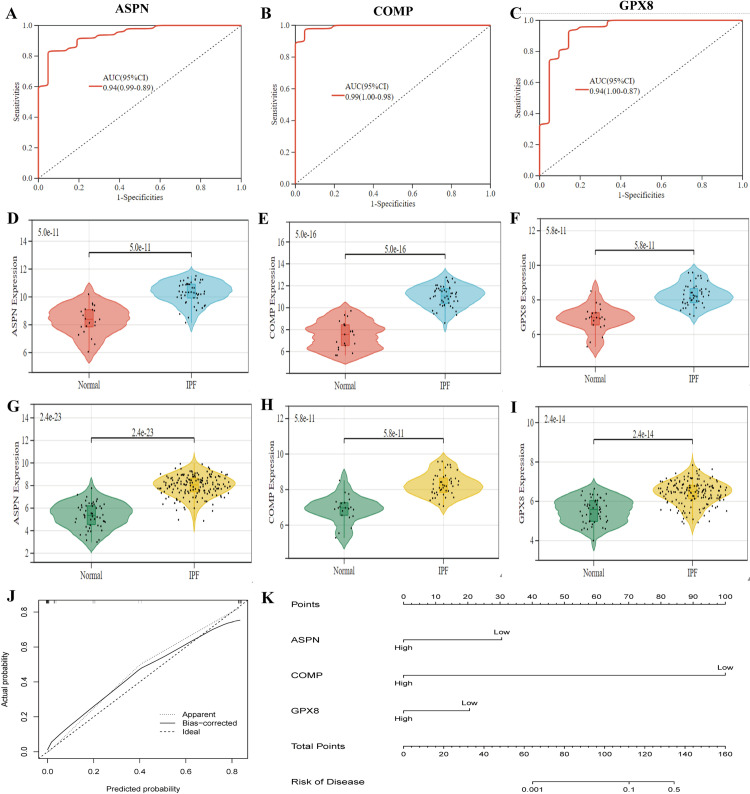
Expression levels of ASPN, COMP, and GPX8. (A-C) ROC curves for ASPN, COMP, and GPX8 in train set. (D-F) Gene expression levels of ASPN, COMP, and GPX8 in the train set. (G-I) Expression levels of ASPN, COMP, and GPX8 in the test set. (J-K) Calibration curves and Nomogram based on the expression levels of ASPN, COMP, and GPX8.

We generated ROC curves to assess the diagnostic performance of the 3 genes: ASPN, COMP, and GPX8. The AUC for ASPN, COMP, and GPX8 were 0.94, 0.99, and 0.94, respectively, indicating excellent diagnostic efficacy for all 3 genes (Fig 4A and 4C). Next, we evaluated the levels of these genes in the training group. As illustrated in Fig 4D and 4F, the levels of ASPN, COMP, and GPX8 were significantly elevated in the IPF group compared to the control group. Additionally, we observed a similar trend in gene levels in the test group, consistent with the experimental group (Fig 4G and 4I). Therefore, we developed a nomogram and calibration curves based on ASPN, COMP, and GPX8 (Fig 4J and 4K).

### Transcriptome analysis and RT-qPCR validation

**Fig 5:**
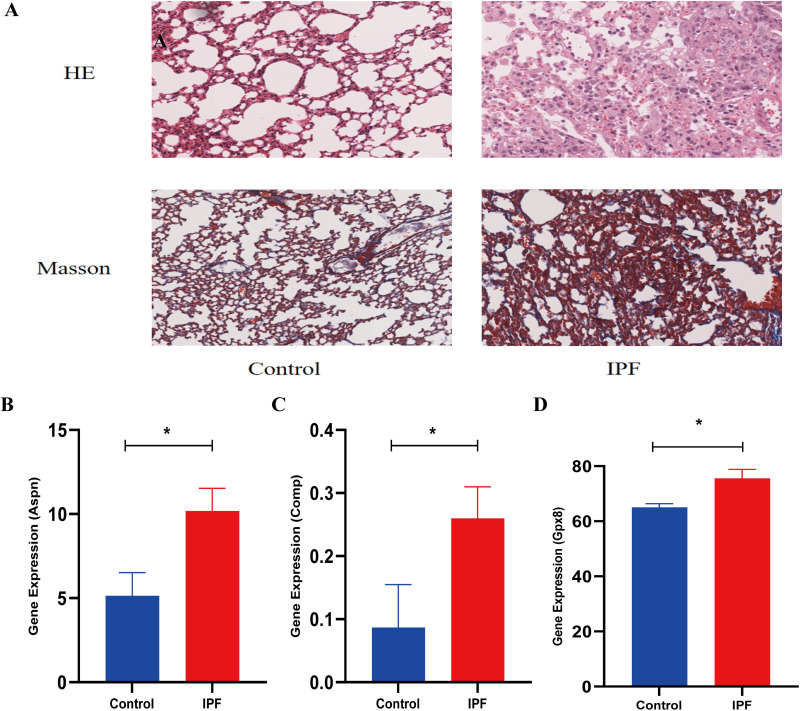
Transcriptomic results of Aspn, Comp, and Gpx8. (A) HE and Masson staining in the control group and the IPF group; (B-D) Gene expression levels of Aspn, Comp, and Gpx8 of control and IPF mice transcriptome analysis. B: Aspn; C: Comp; D: Gpx8.

**Fig 6:**
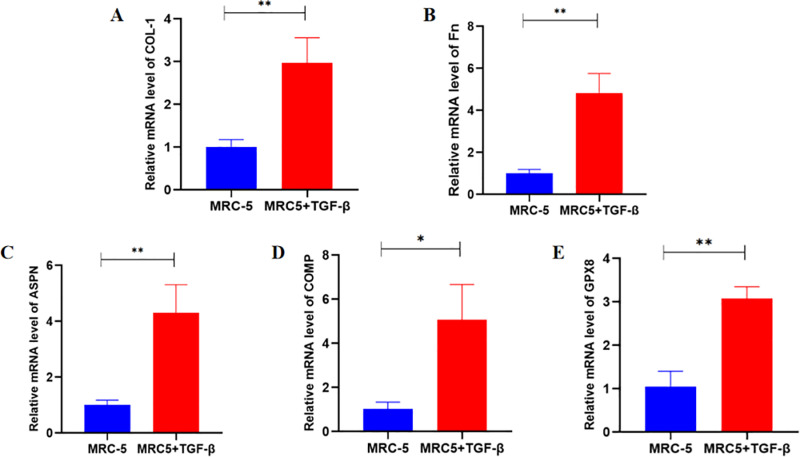
mRNA expression of COL-1, Fn, ASPN, COMP and GPX8 between control group and fibrosis group. (A) COL-1; (B) Fn; (C) ASPN; (D) COMP; (E) GPX8. (^*^P < 0.05, ^**^P < 0.01).

Transcriptome analysis revealed that ASPN, COMP, and GPX8 were highly expressed in BLM treated C57BL/6J mice (Fig 5A-D and S3 Table). RT-qPCR was employed to ascertain the expression profiles of three pivotal genes within MRC5 cells subjected to TGF-β1 treatment. Our initial assessment targeted the expression levels of COL-1 and Fn, confirming the efficacy of our fibrosis-inducing model as depicted in Fig 6A and 6B. Further analysis was conducted on ASPN, COMP, and GPX8, revealing an upward trend in their expression levels within the context of our pulmonary fibrosis cellular model, as illustrated in Fig 6C and 6E.

### Relationship between candidate biomarkers and immune cells

**Fig 7:**
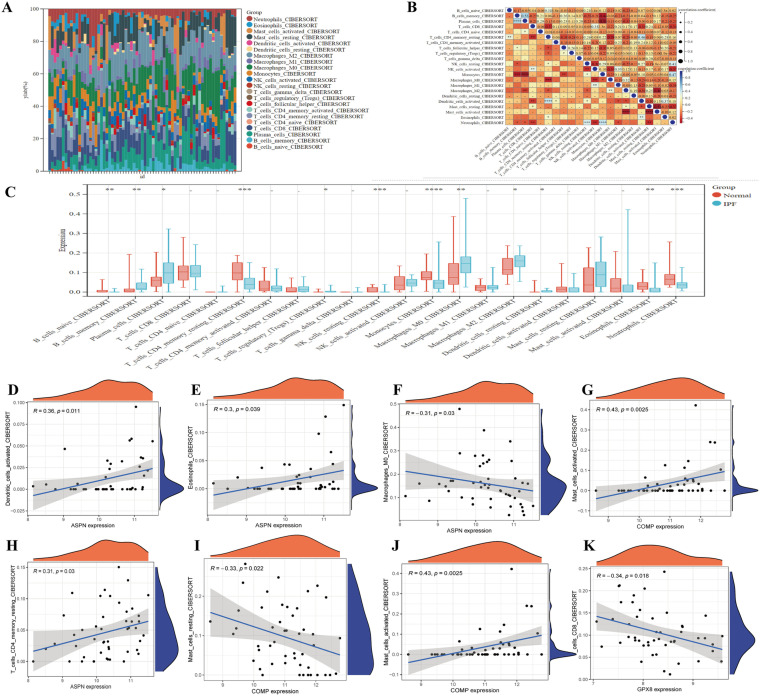
Correlation between candidate biomarkers and immune cells. (A) Stacked bar plot of various immune cells between IPF and normal groups. (B) Heatmap displaying the correlation coefficients among different immune cell types. (C) Boxplot comparing the abundance of various immune cells between IPF and normal groups (*P < 0.05, **P < 0.01, ***P < 0.001). (D-K) Correlation scatter plot illustrating the correlation between the expression levels of candidate biomarkers (ASPN, COMP, GPX8) and specific immune cell types.

We performed immune infiltration analysis using CIBERSORT, revealing an imbalance in immune cells in IPF compared to the normal group (Fig 7A). The correlation heatmap analysis among different immune cell types indicated strong interactions between certain immune cell populations (Fig 7B). We then compared the abundance of different immune cells between the IPF and normal groups, noting significant changes in specific immune cell populations associated with IPF (Fig 7C) Increased neutrophils and activated T cells, coupled with decreased resting memory T cells, M1 and M2 macrophages, and activated NK cells, indicate a complex immune dysregulation involving inflammatory and fibrotic processes. Fig 7D-K present scatter plots showing the correlation between the expression of candidate biomarkers (ASPN, COMP, GPX8) and various immune cells. Fig 7D reveals a strong association between ASPN expression and the abundance of resting dendritic cells(*r* = 0.36, *P* = 0.011), while Fig 7I demonstrates a negative correlation between COMP expression and M2 macrophages (*r* = -0.33, *P* = 0.022). These findings indicate that the expression of certain biomarkers is strongly linked to the presence and activity of specific immune cells in IPF, highlighting the intricate relationship between candidate biomarkers and immune cell populations involved in the disease’s immune mechanisms.

### GSEA and drug sensitivity analysis of candidate biomarkers

**Fig 8:**
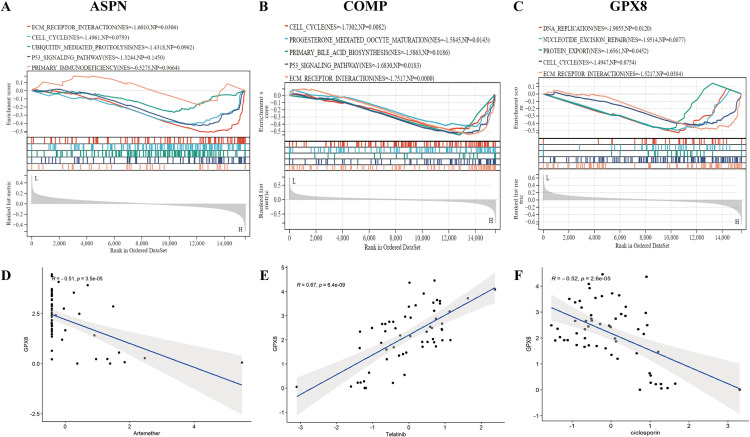
GSEA and drug sensitivity analysis for candidate genes. (A-C) GSEA analysis for ASPN. (B) GSEA analysis for COMP. (C) GSEA analysis for GPX8. (D-F) Drug sensitivity analysis for GPX8.

Next, we performed GSEA and drug sensitivity analysis. Fig 8A-C display the GSEA results for ASPN, COMP, and GPX8, revealing their enrichment in various biological pathways. ASPN is enriched in extracellular matrix receptor interactions and cell cycle pathways (Fig 8A). COMP is enriched in cell cycle and P53 signaling pathways (Fig 8B). GPX8 is enriched in DNA replication and nucleotide excision repair pathways (Fig 8C). Fig 8D-F show the drug sensitivity analysis for GPX8, indicating significant correlations between GPX8 expression and sensitivity to artemether (Fig 8D), telatinib (Fig 8E), and ciclosporin (Fig 8F).

### Analysis of single cell RNA sequencing data

**Fig 9:**
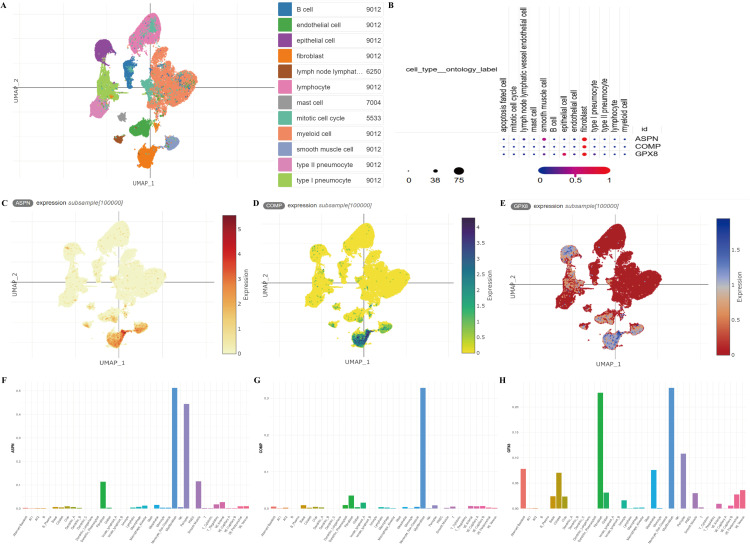
Single-cell sequencing analysis (A) UMAP plot of single-cell RNA sequencing data from human lung tissue. (B) Expression levels of ASPN, COMP, and GPX8 across different cell types. (C)UMAP plot showing the expression of ASPN among cells. (D) UMAP plot showing the expression of COMP among cells. (E) UMAP plot showing the expression of GPX8 among cells. (F) Expression levels of ASPN across different cell types; (G) Expression levels of COMP across different cell types; (H) Expression levels of GPX8 across different cell types.

The single-cell transcriptomic data comprised 716,074 cells and 19,680 genes. Fig 9A shows the UMAP projection of the single-cell transcriptomic data, with cells colored by their identified cell types. The major cell types identified include epithelial cells, fibroblasts, endothelial cells, myeloid cells, lymphocytes, and several other cell types relevant to lung tissue and fibrosis. Fig 9B summarizes the expression levels of ASPN, COMP, and GPX8 across different cell types, highlighting the prominent expression in fibroblasts. Next, we observed high levels of ASPN expression in fibroblasts, indicating its role in the fibrotic process (Fig 9C). Similar to ASPN, COMP is predominantly expressed in fibroblasts, further supporting the involvement of these cells in fibrosis (Fig 9D). The expression pattern of GPX8 is shown in Fig 9E, with GPX8 expression observed across various cell types, notably in epithelial cells and fibroblasts. Analysis based on the IPF Cell Atlas also confirmed that ASPN, COMP, and GPX8 are predominantly expressed in fibroblasts (Fig 9F-H).

### Construction of an ANN Model

**Fig 10:**
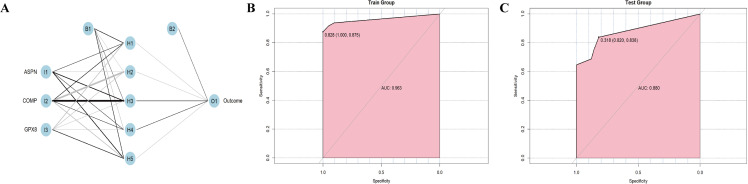
Constructing an artificial neural network model. (A) ANN model for IPF. (B) The training group verifies the ROC curve findings. (C) The testing group verifies the ROC curve findings.

We constructed an artificial neural network (ANN) model based on the gene score table (S4 Table) to classify gene expression data. The ANN model included three input layers, five hidden layers, and one output layer (Fig 10A). Within the training dataset, the AUC metric achieved a value of 0.963, demonstrating the model’s reliability (Fig 10B). Furthermore, we conducted an analysis of the test dataset, resulting in a ROC value of 0.880 (Fig 10C).

## Discussion

IPF is a chronic, progressive interstitial lung disease(ILD) marked by irreversible scarring of lung tissue, resulting in a gradual decline in lung function. The exact cause of IPF remains unknown. Currently, diagnosis of IPF primarily relies on radiological and histological examinations. However, these methods often detect lesions only when the disease has significantly progressed, missing the optimal treatment window. Early diagnosis and disease monitoring can be achieved through screening and identification of reliable biomarkers, thereby improving patient survival and quality of life. Therefore, the search for and test of IPF biomarkers holds significant clinical importance. In this study, we developed a neural network model for IPF diagnosis based on bioinformatics and machine learning techniques. We further investigated the immune infiltration characteristics associated with IPF.

**Fig 11:**
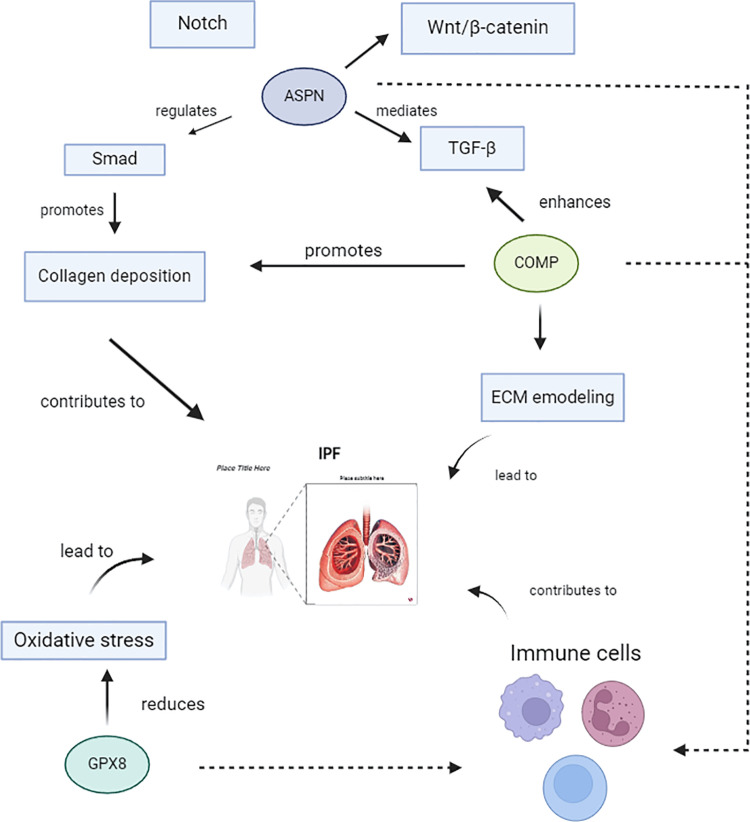
Diagram of the mechanism by which ASPN, COMP, and GPX8 regulate IPF.

In this research, we initially conducted a preliminary examination of 451 DEGs to identify variations between the IPF group and the control group. We then identified IPF-related module genes and ultimately obtained 60 intersecting DEGs. The enrichment analysis uncovered that these genes participate in a multitude of immune-related signaling pathways, underscoring the pivotal role that immune reactions have in the development of IPF. Subsequently, using Lasso regression and RF machine learning methods, We pinpointed 3 key genes: ASPN, COMP, and GPX8. These genes play crucial roles in fibrotic diseases (**Fig 11**).

ASPN (Asporin) is a protein of the extracellular matrix that is part of the small leucine-rich proteoglycan family, playing a crucial role in tissue injury and regeneration [21]. Research indicates that ASPN facilitates epithelial-mesenchymal transition by interacting with various signaling pathways, including TGF-β, Wnt/β-catenin, and Notch, which in turn promotes tissue fibrosis [22,23]. Pulmonary fibrosis is marked by the proliferation of fibroblasts and an abnormal accumulation of extracellular matrix components. Studies suggest that reducing ASPN levels can impede the abnormal differentiation of myofibroblasts and decrease the in vitro expression of pro-fibrotic genes. ASPN regulates the synthesis of extracellular matrix and collagen deposition through the TGF-β/Smad signaling pathway, thereby promoting progression of pulmonary fibrosis [22]. Fibroblasts are highly metabolically active mesenchymal cells that play an essential role in regulating the extracellular matrix during tissue fibrosis. High expression of ASPN in reactive fibroblasts suggests its potential as a key proximal regulator of fibroblast pro-fibrotic signaling [24]. The immune system is pivotal in the development and advancement of IPF, characterized by uncontrolled immune responses and an imbalanced injury-inflammation-repair cycle [25]. Recent studies indicate correlations between ASPN and macrophages, T cells, dendritic cells, etc. Macrophages are crucial for modulating local microenvironments and orchestrating immune responses. Lung macrophages are significant producers of fibrogenic cytokines, chemokines, and proteases. Regulators of M2 macrophage polarization promote the development of pulmonary fibrosis, while inhibiting M2 macrophages is an important strategy for improving pulmonary fibrosis [26,27]. In studies of atherosclerosis, ASPN has been identified as a key gene linked to the polarization of M2 macrophages [28]. In IPF patients, CD4+ T cells are found in alveolar walls, interstitium, and perivascular areas, and are associated with worsening lung function and poorer survival rates [27]. Dendritic cells are antigen-presenting cells unique to the human and mammalian immune systems capable of activating naïve T cells, responsible for both innate and adaptive immunity. Single-cell RNA sequencing data from patients with IPF reveal a substantial increase in both lung macrophages and dendritic cells [29]. ASPN could be a promising therapeutic target for modulating immune responses in IPF.

COMP (Cartilage oligomeric matrix protein), a member of the thrombospondin (TSP) gene family, is a key extracellular matrix protein essential for the assembly of collagen [30,31] (including collagen II and IX, matrilin, etc.) and extracellular matrix stability [32,33]. Excessive matrix deposition in fibrosis can lead to cellular dysfunction, tissue damage, and organ failure. The role of COMP in IPF involves multiple mechanisms. Firstly, increased expression of COMP can enhance TGFβ1 signaling and promote fibrosis by reorganizing extracellular matrix aggregation [34]. Secondly, COMP is essential not only for the assembly of macromolecules within the extracellular matrix but also for directly supporting intracellular collagen secretion [35]. The GEO database shows that COMP is one of the most upregulated genes in IPF, and its elevated levels can serve as a biomarker for various fibrotic diseases [32]. Compared to healthy individuals, IPF patients show significantly increased serum levels of COMP, which correlate with a decline in lung function over time [34]. Immune infiltration results from this study suggest that COMP may also be involved in modulating mast cells. Previous studies have identified a notable rise in the quantity and activity of mast cells within lung tissue affected by IPF in humans, which correlates negatively with lung function. Mast cells accumulate in fibrotic areas of the lungs. Furthermore, a positive feedback mechanism between fibroblasts and mast cells has been identified, which collectively exacerbates tissue damage and remodeling in IPF [36,37]. Proteomic and transcriptomic sequencing results from human IPF samples reveal a marked increase in mast cell chemokines, including CCL5, CCL21, and CXCL12, as well as mast cell protease-2. Among these, CXCL12 may serve as a potential biomarker for advanced stages of IPF [36,38]. Recent studies suggest that nintedanib may exert anti-fibrotic effects by inhibiting mast cell survival and activity [39].

Oxidative stress is pivotal in pulmonary fibrosis, driving recurrent damage to lung epithelial cells, thereby advancing the fibrotic process and compromising lung function [40]. The glutathione peroxidase (GPX) family alleviates oxidative stress by lowering intracellular reactive oxygen species levels, thus influencing the progression of IPF [41]. GPX8 (Glutathione peroxidase 8), an entity within the glutathione peroxidase family, protects cells from damage by combating oxidative stress through multiple pathways [42,43]. Studies indicate that GPX8 expression is influenced by epithelial-mesenchymal transition programs [44]. Studies indicate that GPX8 levels is influenced by epithelial mesenchymal transition programs [45]. GPX8 may also participate in regulating the fibrosis process by influencing immune cell function. GPX8-deficient macrophages show a significant increase in IL-1β production under LPS stimulation, and C57BL/6 mice that received transplants of GPX8-deficient macrophages displayed a phenotype indicative of exacerbated colitis [46].

### Limitations

However, this study has certain limitations. Due to the relative rarity of IPF, the sample size used in this study was relatively small. In addition, the interpretability of the model is also limited. Although the neural network model demonstrated good predictive accuracy, its “black-box” nature makes the prediction results difficult to explain. Third, the interaction data from the STRING database used in the analysis come from various bioinformatics prediction methods and literature mining, which may contain some uncertainty. For example, some interactions may be inferred based on homology or prediction models rather than direct experimental validation. Additionally, GO enrichment analysis assumes that genes are independent, but in reality, there are complex interactions between genes. KEGG pathway analysis relies on known metabolic and signaling pathways, and may not provide accurate annotations for biological processes that have not been sufficiently studied.

### Future research directions

Future studies should focus on overcoming the limitations of the current research and explore the following promising directions: First, large-scale multi-center studies should validate the clinical utility of identified biomarkers (such as GPX8, COMP, and ASPN) in different IPF populations to determine their potential as diagnostic tools. Second, functional validation and exploration of therapeutic targets should be conducted through in vitro and in vivo experiments to verify the function of key genes (such as GPX8, COMP, and ASPN) and explore their potential as therapeutic targets. Finally, novel therapeutic strategies targeting these genes and their associated pathways should be explored.

## Conclusion

This study utilized a neural network diagnostic model to identify GPX8, COMP, and ASPN as key genes in IPF. We extensively explored the roles of these genes in IPF and their relationships with the immune system. Our findings not only provide new potential biomarkers for early diagnosis of IPF but also provide novel perspectives on the underlying pathological mechanisms of IPF. These discoveries could facilitate the development of novel therapeutic strategies for IPF. Future research should further validate the specific mechanisms of these genes in IPF and investigate their potential as therapeutic targets. Additionally, optimizing the model and exploring more immune markers may facilitate earlier and more accurate diagnosis and treatment of IPF.

## Supporting information

S1 Table 451 DEGs of IPF compared to normal group. (XLSX)

S2 Table Genes in the black module of WGCNA. (XLSX)

S3 Table Gene expression levels by transcriptome sequencing. (XLSX)

S4 Table ASPN, COMP, and GPX8 gene in the train set. (XLSX)

## References

[1] Wuyts WA, Wijsenbeek M, Bondue B, Bouros D, Bresser P, Robalo Cordeiro C, et al. Idiopathic pulmonary fibrosis: best practice in monitoring and managing a relentless fibrotic disease. Respiration. 2020;99(1):73–82. doi: 10.1159/000504763 31830755 PMC6979429

[2] Raghu G, Collard HR, Egan JJ, Martinez FJ, Behr J, Brown KK, et al. An official ATS/ERS/JRS/ALAT statement: idiopathic pulmonary fibrosis: evidence-based guidelines for diagnosis and management. Am J Respir Crit Care Med. 2011;183(6):788–824. doi: 10.1164/rccm.2009-040GL 21471066 PMC5450933

[3] Raghu G, Remy-Jardin M, Myers JL, Richeldi L, Ryerson CJ, Lederer DJ, et al. Diagnosis of idiopathic pulmonary fibrosis. An Official ATS/ERS/JRS/ALAT clinical practice guideline. Am J Respir Crit Care Med. 2018;198(5):e44–68. doi: 10.1164/rccm.201807-1255ST 30168753

[4] Lynch DA, Sverzellati N, Travis WD, Brown KK, Colby TV, Galvin JR, et al. Diagnostic criteria for idiopathic pulmonary fibrosis: a Fleischner society white paper. Lancet Respir Med. 2018;6(2):138–53. doi: 10.1016/S2213-2600(17)30433-2 29154106

[5] Topol EJ. High-performance medicine: the convergence of human and artificial intelligence. Nat Med. 2019;25(1):44–56. doi: 10.1038/s41591-018-0300-7 30617339

[6] Mutsaers SE, Miles T, Prêle CM, Hoyne GF. Emerging role of immune cells as drivers of pulmonary fibrosis. Pharmacol Ther. 2023;252:108562. doi: 10.1016/j.pharmthera.2023.108562 37952904

[7] Heukels P, Moor CC, von der Thüsen JH, Wijsenbeek MS, Kool M. Inflammation and immunity in IPF pathogenesis and treatment. Respir Med. 2019;147:79–91. doi: 10.1016/j.rmed.2018.12.015 30704705

[8] Wynn TA, Vannella KM. Macrophages in tissue repair, regeneration, and fibrosis. Immunity. 2016;44(3):450–62. doi: 10.1016/j.immuni.2016.02.015 26982353 PMC4794754

[9] Johnson WE, Li C, Rabinovic A. Adjusting batch effects in microarray expression data using empirical Bayes methods. Biostatistics. 2007;8(1):118–27. doi: 10.1093/biostatistics/kxj037 16632515

[10] Bolstad BM, Irizarry RA, Astrand M, Speed TP. A comparison of normalization methods for high density oligonucleotide array data based on variance and bias. Bioinformatics. 2003;19(2):185–93. doi: 10.1093/bioinformatics/19.2.185 12538238

[11] Ritchie ME, Phipson B, Wu D, Hu Y, Law CW, Shi W, et al. limma powers differential expression analyses for RNA-sequencing and microarray studies. Nucleic Acids Res. 2015;43(7):e47. doi: 10.1093/nar/gkv007 25605792 PMC4402510

[12] Langfelder P, Horvath S. WGCNA: an R package for weighted correlation network analysis. BMC Bioinformatics. 2008;9:559. doi: 10.1186/1471-2105-9-559 19114008 PMC2631488

[13] Yu G, Wang L-G, Han Y, He Q-Y. clusterProfiler: an R package for comparing biological themes among gene clusters. OMICS. 2012;16(5):284–7. doi: 10.1089/omi.2011.0118 22455463 PMC3339379

[14] Yu G, Wang L-G, Yan G-R, He Q-Y. DOSE: an R/Bioconductor package for disease ontology semantic and enrichment analysis. Bioinformatics. 2015;31(4):608–9. doi: 10.1093/bioinformatics/btu684 25677125

[15] Chen X, Ishwaran H. Random forests for genomic data analysis. Genomics. 2012;99(6):323–9. doi: 10.1016/j.ygeno.2012.04.003 22546560 PMC3387489

[16] Friedman J, Hastie T, Tibshirani R. Regularization paths for generalized linear models via coordinate descent. J Stat Softw. 2010;33(1):1–22. doi: 10.18637/jss.v033.i01 20808728 PMC2929880

[17] Shen W, Song Z, Zhong X, Huang M, Shen D, Gao P, et al. Sangerbox: A comprehensive, interaction-friendly clinical bioinformatics analysis platform. Imeta. 2022;1(3):e36. doi: 10.1002/imt2.36 38868713 PMC10989974

[18] Liberzon A, Subramanian A, Pinchback R, Thorvaldsdóttir H, Tamayo P, Mesirov JP. Molecular signatures database (MSigDB) 3.0. Bioinformatics. 2011;27(12):1739–40. doi: 10.1093/bioinformatics/btr260 21546393 PMC3106198

[19] Reinhold WC, Sunshine M, Liu H, Varma S, Kohn KW, Morris J, et al. CellMiner: a web-based suite of genomic and pharmacologic tools to explore transcript and drug patterns in the NCI-60 cell line set. Cancer Res. 2012;72(14):3499–511. doi: 10.1158/0008-5472.CAN-12-1370 22802077 PMC3399763

[20] Beck MW. NeuralNetTools: visualization and analysis tools for neural networks. J Stat Softw. 2018;85(11):1–20. doi: 10.18637/jss.v085.i11 30505247 PMC6262849

[21] Henry SP, Takanosu M, Boyd TC, Mayne PM, Eberspaecher H, Zhou W, et al. Expression pattern and gene characterization of asporin. a newly discovered member of the leucine-rich repeat protein family. J Biol Chem. 2001;276(15):12212–21. doi: 10.1074/jbc.M011290200 11152695

[22] Huang S, Lai X, Yang L, Ye F, Huang C, Qiu Y, et al. Asporin promotes TGF-β-induced lung myofibroblast differentiation by facilitating Rab11-dependent recycling of TβRI. Am J Respir Cell Mol Biol. 2022;66(2):158–70. doi: 10.1165/rcmb.2021-0257OC 34705621

[23] Lall SP, Alsafwani ZW, Batra SK, Seshacharyulu P. ASPORIN: a root of the matter in tumors and their host environment. Biochim Biophys Acta Rev Cancer. 2024;1879(1):189029. doi: 10.1016/j.bbcan.2023.189029 38008263 PMC10872503

[24] Huang C, Sharma A, Thakur R, Rai D, Katiki M, Germano J de F, et al. Asporin, an extracellular matrix protein, is a beneficial regulator of cardiac remodeling. Matrix Biol. 2022;110:40–59. doi: 10.1016/j.matbio.2022.04.005 35470068 PMC10234622

[25] van Geffen C, Deißler A, Quante M, Renz H, Hartl D, Kolahian S. Regulatory immune cells in idiopathic pulmonary fibrosis: friends or foes?. Front Immunol. 2021;12:663203. doi: 10.3389/fimmu.2021.663203 33995390 PMC8120991

[26] Cheng P, Li S, Chen H. Macrophages in lung injury, repair, and fibrosis. Cells. 2021;10(2):436. doi: 10.3390/cells10020436 33670759 PMC7923175

[27] Parra ER, Kairalla RA, Ribeiro de Carvalho CR, Eher E, Capelozzi VL. Inflammatory cell phenotyping of the pulmonary interstitium in idiopathic interstitial pneumonia. Respiration. 2007;74(2):159–69. doi: 10.1159/000097133 17108669

[28] Yuan Y, Wang P, Zhang H, Liu Y. Identification of M2 macrophage-related key genes in advanced atherosclerotic plaques by network-based analysis. J Cardiovasc Pharmacol. 2024;83(3):276–88. doi: 10.1097/FJC.0000000000001528 38194604

[29] Serezani APM, Pascoalino BD, Bazzano JMR, Vowell KN, Tanjore H, Taylor CJ, et al. Multiplatform single-cell analysis identifies immune cell types enhanced in pulmonary fibrosis. Am J Respir Cell Mol Biol. 2022;67(1):50–60. doi: 10.1165/rcmb.2021-0418OC 35468042 PMC9273229

[30] Heinegård D. Fell-Muir Lecture: proteoglycans and more--from molecules to biology. Int J Exp Pathol. 2009;90(6):575–86. doi: 10.1111/j.1365-2613.2009.00695.x 19958398 PMC2803248

[31] Hecht JT, Hayes E, Haynes R, Cole WG. COMP mutations, chondrocyte function and cartilage matrix. Matrix Biol. 2005;23(8):525–33. doi: 10.1016/j.matbio.2004.09.006 15694129

[32] Agarwal P, Schulz J-N, Blumbach K, Andreasson K, Heinegård D, Paulsson M, et al. Enhanced deposition of cartilage oligomeric matrix protein is a common feature in fibrotic skin pathologies. Matrix Biol. 2013;32(6):325–31. doi: 10.1016/j.matbio.2013.02.010 23507196

[33] Moinzadeh P, Agarwal P, Bloch W, Orteu C, Hunzelmann N, Eckes B, et al. Systemic sclerosis with multiple nodules: characterization of the extracellular matrix. Arch Dermatol Res. 2013;305(7):645–52. doi: 10.1007/s00403-013-1383-0 23836353

[34] Vuga LJ, Milosevic J, Pandit K, Ben-Yehudah A, Chu Y, Richards T, et al. Cartilage oligomeric matrix protein in idiopathic pulmonary fibrosis. PLoS One. 2013;8(12):e83120. doi: 10.1371/journal.pone.0083120 24376648 PMC3869779

[35] Schulz J-N, Plomann M, Sengle G, Gullberg D, Krieg T, Eckes B. New developments on skin fibrosis - Essential signals emanating from the extracellular matrix for the control of myofibroblasts. Matrix Biol. 2018;68–69:522–32. doi: 10.1016/j.matbio.2018.01.025 29408278

[36] Wygrecka M, Dahal BK, Kosanovic D, Petersen F, Taborski B, von Gerlach S, et al. Mast cells and fibroblasts work in concert to aggravate pulmonary fibrosis: role of transmembrane SCF and the PAR-2/PKC-α/Raf-1/p44/42 signaling pathway. Am J Pathol. 2013;182(6):2094–108. doi: 10.1016/j.ajpath.2013.02.013 23562441

[37] Savage A, Risquez C, Gomi K, Schreiner R, Borczuk AC, Worgall S, et al. The mast cell exosome-fibroblast connection: a novel pro-fibrotic pathway. Front Med (Lausanne). 2023;10:1139397. doi: 10.3389/fmed.2023.1139397 36910476 PMC9995661

[38] Sivakumar P, Ammar R, Thompson JR, Luo Y, Streltsov D, Porteous M, et al. Integrated plasma proteomics and lung transcriptomics reveal novel biomarkers in idiopathic pulmonary fibrosis. Respir Res. 2021;22(1):273. doi: 10.1186/s12931-021-01860-3 34689792 PMC8543878

[39] Overed-Sayer C, Miranda E, Dunmore R, Liarte Marin E, Beloki L, Rassl D, et al. Inhibition of mast cells: a novel mechanism by which nintedanib may elicit anti-fibrotic effects. Thorax. 2020;75(9):754–63. doi: 10.1136/thoraxjnl-2019-214000 32709610 PMC7476277

[40] Estornut C, Milara J, Bayarri MA, Belhadj N, Cortijo J. Targeting oxidative stress as a therapeutic approach for idiopathic pulmonary fibrosis. Front Pharmacol. 2022;12:794997. doi: 10.3389/fphar.2021.794997 35126133 PMC8815729

[41] Fois AG, Paliogiannis P, Sotgia S, Mangoni AA, Zinellu E, Pirina P, et al. Evaluation of oxidative stress biomarkers in idiopathic pulmonary fibrosis and therapeutic applications: a systematic review. Respir Res. 2018;19(1):51. doi: 10.1186/s12931-018-0754-7 29587761 PMC5872514

[42] Yoboue ED, Rimessi A, Anelli T, Pinton P, Sitia R. Regulation of calcium fluxes by GPX8, a Type-II transmembrane peroxidase enriched at the mitochondria-associated endoplasmic reticulum membrane. Antioxid Redox Signal. 2017;27(9):583–95. doi: 10.1089/ars.2016.6866 28129698

[43] Bosello Travain V, Miotto G, Vučković A-M, Cozza G, Roveri A, Toppo S, et al. Lack of glutathione peroxidase-8 in the ER impacts on lipid composition of HeLa cells microsomal membranes. Free Radic Biol Med. 2020;147:80–9. doi: 10.1016/j.freeradbiomed.2019.12.010 31857233

[44] Khatib A, Solaimuthu B, Ben Yosef M, Abu Rmaileh A, Tanna M, Oren G, et al. The glutathione peroxidase 8 (GPX8)/IL-6/STAT3 axis is essential in maintaining an aggressive breast cancer phenotype. Proc Natl Acad Sci U S A. 2020;117(35):21420–31. doi: 10.1073/pnas.2010275117 32817494 PMC7474634

[45] Piñeiro-Hermida S, López IP, Alfaro-Arnedo E, Torrens R, Iñiguez M, Alvarez-Erviti L, et al. IGF1R deficiency attenuates acute inflammatory response in a bleomycin-induced lung injury mouse model. Sci Rep. 2017;7(1):4290. doi: 10.1038/s41598-017-04561-4 28655914 PMC5487362

[46] Hsu J-L, Chou J-W, Chen T-F, Hsu J-T, Su F-Y, Lan J-L, et al. Glutathione peroxidase 8 negatively regulates caspase-4/11 to protect against colitis. EMBO Mol Med. 2020;12(1):e9386. doi: 10.15252/emmm.201809386 31782617 PMC6949489

